# Construction and validation of a nomogram to predict the overall survival of small cell lung cancer: a multicenter retrospective study in Shandong province, China

**DOI:** 10.1186/s12885-023-11692-7

**Published:** 2023-12-01

**Authors:** Ziqian Song, Hengmin Ma, Hao Sun, Qiuxia Li, Yan Liu, Jing Xie, Yukun Feng, Yuwang Shang, Kena Ma, Nan Zhang, Jialin Wang

**Affiliations:** 1 Shandong Cancer Hospital and Institute, Shandong First Medical University and Shandong Academy of Medical Sciences, Jinan, 250000 China; 2 School of Public Health, Weifang Medical University, Weifang, 261053 China; 3 Centre for Health Management and Policy Research, School of Public Health, Cheeloo College of Medicine, Shandong University, No. 44 Wenhuaxi Road, Jinan, Shandong 250012 China; 4 NHC Key Lab of Health Economics and Policy Research, Shandong University, No. 44 Wenhuaxi Road, Jinan, Shandong 250012 China

**Keywords:** Lung cancer, Clinical characteristics, Prognostic factors, Survival rate, Multi-center

## Abstract

**Background:**

Patients diagnosed with small cell lung cancer (SCLC) typically experience a poor prognosis, and it is essential to predict overall survival (OS) and stratify patients based on distinct prognostic risks.

**Methods:**

Totally 2309 SCLC patients from the hospitals in 15 cities of Shandong from 2010 − 2014 were included in this multicenter, population-based retrospective study. The data of SCLC patients during 2010–2013 and in 2014 SCLC were used for model development and validation, respectively. OS served as the primary outcome. Univariate and multivariate Cox regression were applied to identify the independent prognostic factors of SCLC, and a prognostic model was developed based on these factors. The discrimination and calibration of this model were assessed by the time-dependent C-index, time-dependent receiver operator characteristic curves (ROC), and calibration curves. Additionally, Decision Curve Analysis (DCA) curves, Net Reclassification Improvement (NRI), and Integrated Discriminant Improvement (IDI) were used to assess the enhanced clinical utility and predictive accuracy of the model compared to TNM staging systems.

**Results:**

Multivariate analysis showed that region (Southern/Eastern, hazard ratio [HR] = 1.305 [1.046 − 1.629]; Western/Eastern, HR = 0.727 [0.617 − 0.856]; Northern/Eastern, HR = 0.927 [0.800 − 1.074]), sex (female/male, HR = 0.838 [0.737 − 0.952]), age (46–60/≤45, HR = 1.401 [1.104 − 1.778]; 61–75/≤45, HR = 1.500 [1.182 − 1.902]; >75/≤45, HR = 1.869 [1.382 − 2.523]), TNM stage (II/I, HR = 1.119[0.800 − 1.565]; III/I, HR = 1.478 [1.100 − 1.985]; IV/I, HR = 1.986 [1.477 − 2.670], surgery (yes/no, HR = 0.677 [0.521 − 0.881]), chemotherapy (yes/no, HR = 0.708 [0.616 − 0.813]), and radiotherapy (yes/no, HR = 0.802 [0.702 − 0.917]) were independent prognostic factors of SCLC patients and were included in the nomogram. The time-dependent AUCs of this model in the training set were 0.699, 0.683, and 0.683 for predicting 1-, 3-, and 5-year OS, and 0.698, 0.698, and 0.639 in the validation set, respectively. The predicted calibration curves aligned with the ideal curves, and the DCA curves, the IDI, and the NRI collectively demonstrated that the prognostic model had a superior net benefit than the TNM staging system.

**Conclusion:**

The nomogram using SCLC patients in Shandong surpassed the TNM staging system in survival prediction accuracy and enabled the stratification of patients with distinct prognostic risks based on nomogram scores.

**Supplementary Information:**

The online version contains supplementary material available at 10.1186/s12885-023-11692-7.

## Introduction

Lung cancer is a major public health problem that caused 1.8 million deaths from 2.21 million cases worldwide in 2020 [1]. The incidence rate of lung cancer is high in China, as data from the National Cancer Center (NCC) of China shows 828,000 new cases and 657,000 deaths from lung cancer in 2016 [2]. Small cell lung cancer (SCLC) accounts for approximately 13–15% of new lung cancer cases, and its high rate of invasion has been a primary concern of the medical community for many years [3, 4]. The lack of clear biomarkers for the prediction of SCLC, along with the absence of novel and effective treatments, has made it difficult to improve patient survival rates [5–7]. Pathological staging is correlated with the treatment choice and prognostic value in cancer patients, and the U.S. Veterans Administration Lung Cancer Study Group (VALCSG) broadly classifies SCLC staging into limited and extensive stages [8]. However, the American Joint Committee on Cancer (AJCC) released the 8th edition of lung cancer tumor-lymph node metastasis (TNM) staging in 2015, which provides more detailed information compared to the VALCSG staging system [9]. SCLC is characterized by an abbreviated tumor doubling time, metastasis at an early stage, poor prognosis, and more than half of the patients are diagnosed at an advanced stage, so it is necessary to design a risk model that can predict the overall survival (OS) prognosis of patients [10]. Presently existing prognostic models for SCLC are delimited to either public databases or the confines of singular-center hospitals [11–14]. Their predictive capabilities exhibit inconsistent performance, and a substantial portion remains devoid of external validation.

The nomogram model predicts patient prognosis based on the staging system and incorporates additional risk factors that have been widely used to predict cancer survival [15–17]. For our study, we selected SCLC patients from 186 hospitals in fifteen cities in Shandong for the survey and prognostic follow-up. We statistically analyzed the patients’ clinical characteristics, prognosis, and survival status to identify prognostic influencing factors. We constructed a prognostic nomogram model to predict the probability of patient survival, which could enable clinicians to tailor treatment strategies to individual patient characteristics through meticulous consideration of multiple prognostic factors, optimizing therapeutic interventions. The visual representation of complex prognostic information facilitates clear communication between healthcare providers and patients, empowering the latter to actively participate in informed decision-making.

## Materials and methods

### Patients and variables

This is a multicenter, population-based retrospective study using the database of 25 494 lung cancer patients from 186 tumor hospitals and general hospitals in Shandong between 2010 and 2014 established on the subject “Attributable risk of lung cancer to the disease burden caused by atmospheric pollution” from the National Cancer Center of China. Socio-demographic and clinical information on lung cancer patients were obtained through medical record extracts, and survival information were obtained using a combination of passive and active follow-up. Passive follow-up mainly relied on data from the Total Cause of Death Surveillance System, and we obtained follow-up data by comparing information from the morbidity and mortality pools. In contrast, active follow-up was to proactively obtain patient survival information through household visits or phone calls, with a December 31, 2019 deadline for follow-up. We strictly reviewed each record according to the uniform standards set by the World Health Organization’s International Agency for Cancer Registries (IACR). For cases with errors in coding, gender, date, and other logical errors, we provided immediate feedback and double-check the original data. Data that fail multiple quality control reviews of population tumor follow-up were excluded from the analysis.

**Fig. 1:**
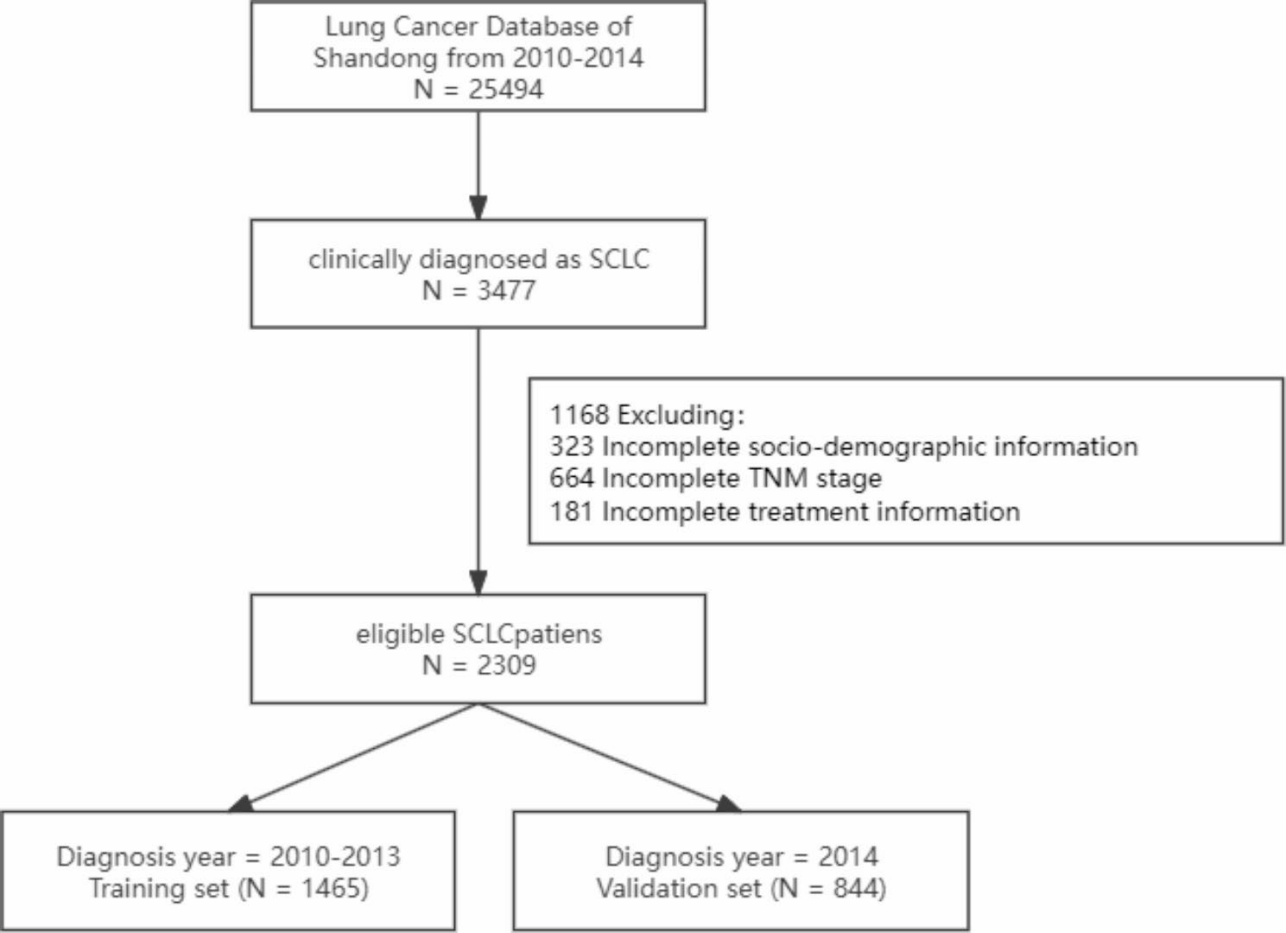
Flowchart of patients with small cell lung cancer screening and dividing. *Note*: socio-demographic information including age, sex, smoking status, alcohol use status, health insurance, region, and attending hospital; treatment information including surgery, chemotherapy, and radiotherapy

Cases of SCLC were diagnosed according to the International Statistical Classification of Diseases and Related Health Problems, Version 10 (ICD-10), code C34, and were confirmed by the cancer register record. We initially screened 3477 SCLC cases from the database, excluding 323 cases without complete socio-demographic characteristics, 664 cases with incomplete TNM stage, and 181 cases with incomplete treatment information (Fig. 1). The selection of prognostic factors was based on previous studies and references [11–13], and the information collected included socio-demographic statistics, such as age, sex, smoking status, alcohol use status, health insurance, region, and attending hospital. Pathological staging was based on the AJCC 8th edition TNM staging of lung cancer, and treatment-related data included information on surgery, chemotherapy, and radiotherapy. Overall survival (OS) was defined as the length of time from diagnosis to death or last exposure. This OS was then used as the primary outcome, with no more than five years of exposure. The study was approved by the National Cancer Center of China and the Shandong Cancer Hospital and Institute.

### Statistical analyses

We divided the dataset to use the 2010 − 2013 SCLC as a training set to develop the model and the 2014 SCLC as a validation set to validate the model. The training set was used for univariate and multivariate Cox proportional hazard regression analyses to identify the factors associated with patient prognosis. We utilized the prognostic factors determined in the multivariate analysis to construct the nomogram and then tested its ability to predict 1-, 3-, and 5-year survival in the training set.

To further study the robustness and reliability of the models, we used the 2014 patients as an external validation dataset by evaluating the agreement of the results between the two datasets, which were temporally and geographically independent of each other. The evaluation of internal and external validation of prognostic models used time-dependent C-index, time-dependent receiver operator characteristic curves (ROC), and calibration curves to assess model discrimination and calibration. We also constructed decision curve analysis (DCA) curves and calculated Integrated Discrimination Improvement (IDI) and Net Reclassification Improvement (NRI) to evaluate the clinical utility of this model.

Moreover, we also conducted a sensitivity analysis by treating age as a continuous variable. All statistical analyses were performed using R software version 4.2.2, using the R packages mainly survival, survminer, rms, timeROC, ggDCA, and survIDINRI. The threshold for significance was P < 0.05 in two-sided tests.

## Results

### Patient characteristics

**Fig. 2:**
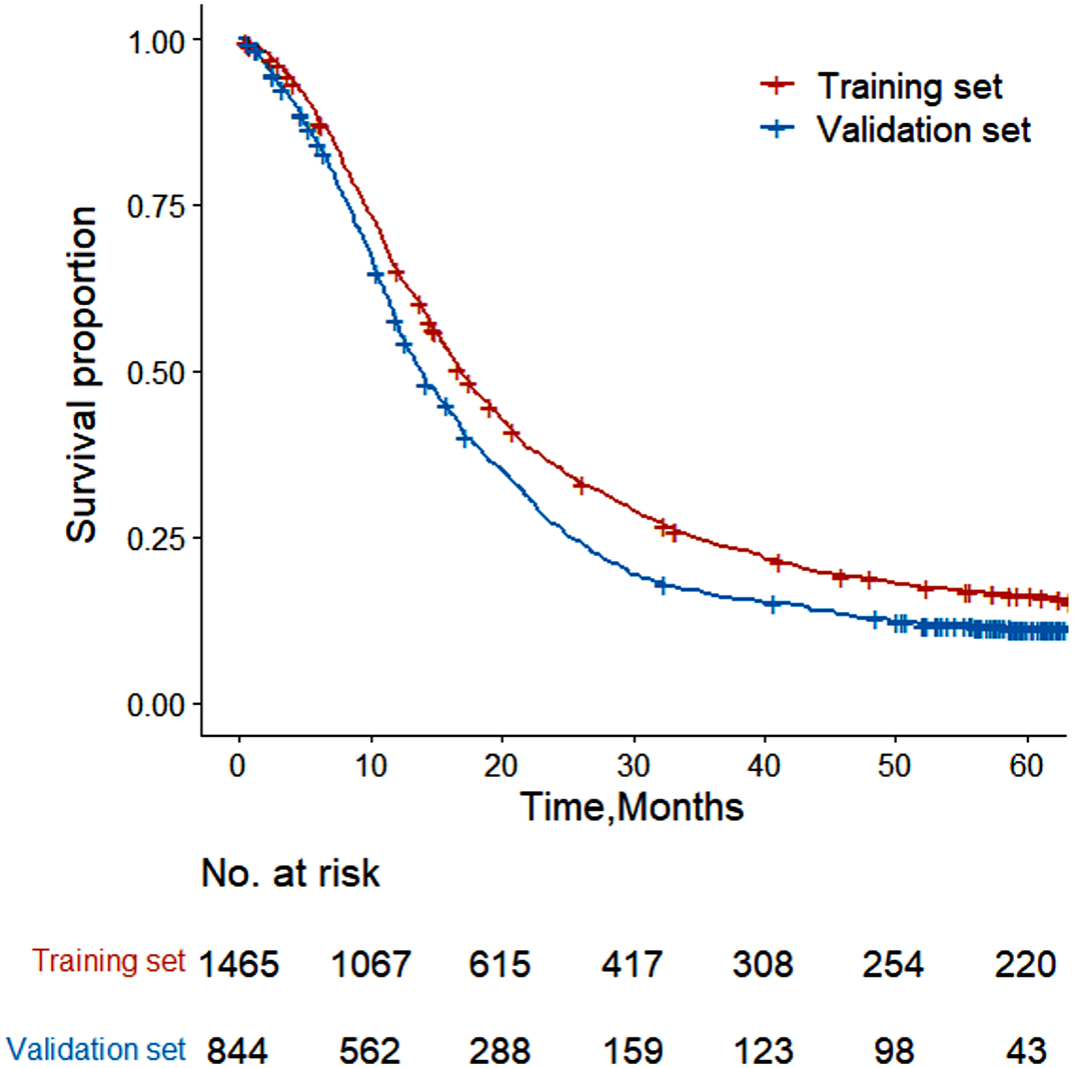
Kaplan–Meier curves for overall survival of patients with small cell lung cancer in the training and validation sets

In this study, a total of 2309 SCLC patients were included, with 1465 participants from 2010 to 2013 in the training set and 844 participants from 2014 in the validation set. Table 1 presents the detailed characteristics of SCLC patients. The overall 5-year survival rate of Shandong SCLC patients was 14.36% with the median survival time of 15.90 months (Fig. 2). The 5-year survival rate in the training set was 16.16%, with a median survival time of 16.87 months while the 5-year survival rate in the validation set was 11.19%, with the median survival time of 13.83 months. The median follow-up was 71.83 months in the whole population, 77.53 months in the training set, and 59.60 months in the validation set.

**Table 1:** Demographic and clinical characteristics of patients with small cell lung cancer in the training set and validation set

Characteristics	Training set, No. (%) (n = 1465)	Validation set, No. (%) (n = 844)	*χ* ^*2*^	*P value*
Sex				
Male	1071 (73.11)	598 (70.85)		
Female	394 (26.89)	246 (29.15)	1.356	0.244
Age				
≤ 45	110 (7.51)	41 (4.86)		
46–60	601 (41.02)	328 (38.86)		
61–75	647 (44.16)	411 (48.70)	8.833	0.032
> 75	107 (7.30)	64 (7.58)		
Region				
Eastern	302 (20.61)	126 (14.93)		
Southern	122 (8.33)	110 (13.03)		
Western	402 (27.44)	331 (39.22)	60.276	< 0.001
Northern	639 (43.62)	277 (32.82)		
Hospital				
Specialized	227 (15.49)	124 (14.69)		
General	1238 (84.51)	720 (85.31)	0.268	0.605
Health insurance				
Urban employees’ basic medical insurance	388 (26.48)	138 (16.35)		
Urban residents’ basic medical insurance	168 (11.47)	141 (16.71)		
New rural cooperative medical scheme	833 (56.86)	506 (59.95)	61.652	< 0.001
Self-pay	56 (3.82)	20 (2.37)		
Other	20 (1.37)	39 (4.62)		
Smoke				
No	582 (39.73)	348 (41.23)		
Yes	883 (60.27)	496 (58.77)	0.504	0.478
Alcohol use				
No	977 (66.69)	555 (65.76)		
Yes	488 (33.31)	289 (34.24)	0.208	0.648
TNM Stage				
I	75 (5.12)	47 (5.57)		
II	141 (9.62)	77 (9.12)		
III	592 (40.41)	331 (39.22)	0.720	0.868
IV	657 (44.85)	389 (46.09)		
Surgery				
No	1373 (93.72)	800 (94.79)		
Yes	92 (6.28)	44 (5.21)	1.099	0.294
Chemotherapy				
No	305 (20.82)	216 (25.59)		
Yes	1160 (79.18)	628 (74.41)	6.983	0.008
Radiotherapy				
No	1095 (74.74)	670 (79.38)		
Yes	370 (25.26)	174 (20.62)	6.401	0.011

*Notes*: Eastern, Southern, Western, and Northern represent respectively Eastern of Shandong, Southern of Shandong, Western of Shandong, and Northern of Shandong; Specialized, General represent respectively specialized tumor hospitals and general hospitals

### Nomogram variable screening

The Cox proportional hazard regression model was performed to conduct the univariate and multivariate analyses in the training set (Table 2). We then selected final variables, including sex, age, region, TNM stage, surgery, chemotherapy, and radiotherapy with a significance threshold of p < 0.05.

**Table 2:** Univariate and multivariate analysis of predictors selected by Cox regression model in the training set

Variables	Univariate analysis			Multivariate analysis	
	*HR (95% CI)*	*P Value*		*HR (95% CI)*	*P Value*
Sex					
Male	Reference			Reference	
Female	0.839 (0.739–0.952)	0.006		0.851 (0.728–0.995)	0.043
Age					
≤ 45	Reference			Reference	
46–60	1.423 (1.122–1.805)	0.004		1.397 (1.100-1.774)	0.006
61–75	1.723 (1.361–2.182)	< 0.001		1.493 (1.175–1.896)	0.001
> 75	2.378 (1.768–3.198)	< 0.001		1.861 (1.376–2.516)	< 0.001
Region					
Eastern	Reference			Reference	
Southern	1.261 (1.011–1.572)	0.039		1.304 (1.045–1.628)	0.019
Western	0.685 (0.583–0.804)	< 0.001		0.726 (0.616–0.855)	< 0.001
Northern	0.909 (0.785–1.052)	0.199		0.926 (0.799–1.073)	0.306
Hospital					
Specialized	Reference				
General	1.032 (0.884–1.204)	0.693			
Health insurance					
Urban employees’ basic medical insurance	Reference				
Urban residents’ basic medical insurance	0.986 (0.810–1.200)	0.885			
New rural cooperative medical scheme	0.972 (0.853–1.108)	0.673			
Self-pay	1.069 (0.794–1.440)	0.660			
Other	0.687 (0.409–1.154)	0.156			
Smoke					
No	Reference			Reference	
Yes	1.159 (1.034–1.298)	0.011		1.025 (0.89–1.182)	0.729
Alcohol use					
No	Reference				
Yes	1.074 (0.955–1.208)	0.231			
TNM Stage					
I	Reference			Reference	
II	1.152 (0.830–1.597)	0.398		1.116 (0.797–1.561)	0.523
III	1.468 (1.108–1.944)	0.007		1.471 (1.094–1.978)	0.011
IV	2.142 (1.620–2.832)	< 0.001		1.977 (1.469–2.66)	< 0.001
Surgery					
No	Reference			Reference	
Yes	0.563 (0.439–0.721)	< 0.001		0.675 (0.519–0.879)	0.003
Chemotherapy					
No	Reference			Reference	
Yes	0.674 (0.589–0.770)	< 0.001		0.707 (0.616–0.813)	< 0.001
Radiotherapy					
No	Reference			Reference	
Yes	0.775 (0.681–0.882)	< 0.001		0.803 (0.702–0.917)	< 0.001

*Notes*: HR, hazard ratio; Eastern, Southern, Western, and Northern represent respectively Eastern of Shandong, Southern of Shandong, Western of Shandong, and Northern of Shandong; Specialized, General represent respectively specialized tumor hospitals and general hospitals

### Construction and validation of the nomogram

**Fig. 3:**
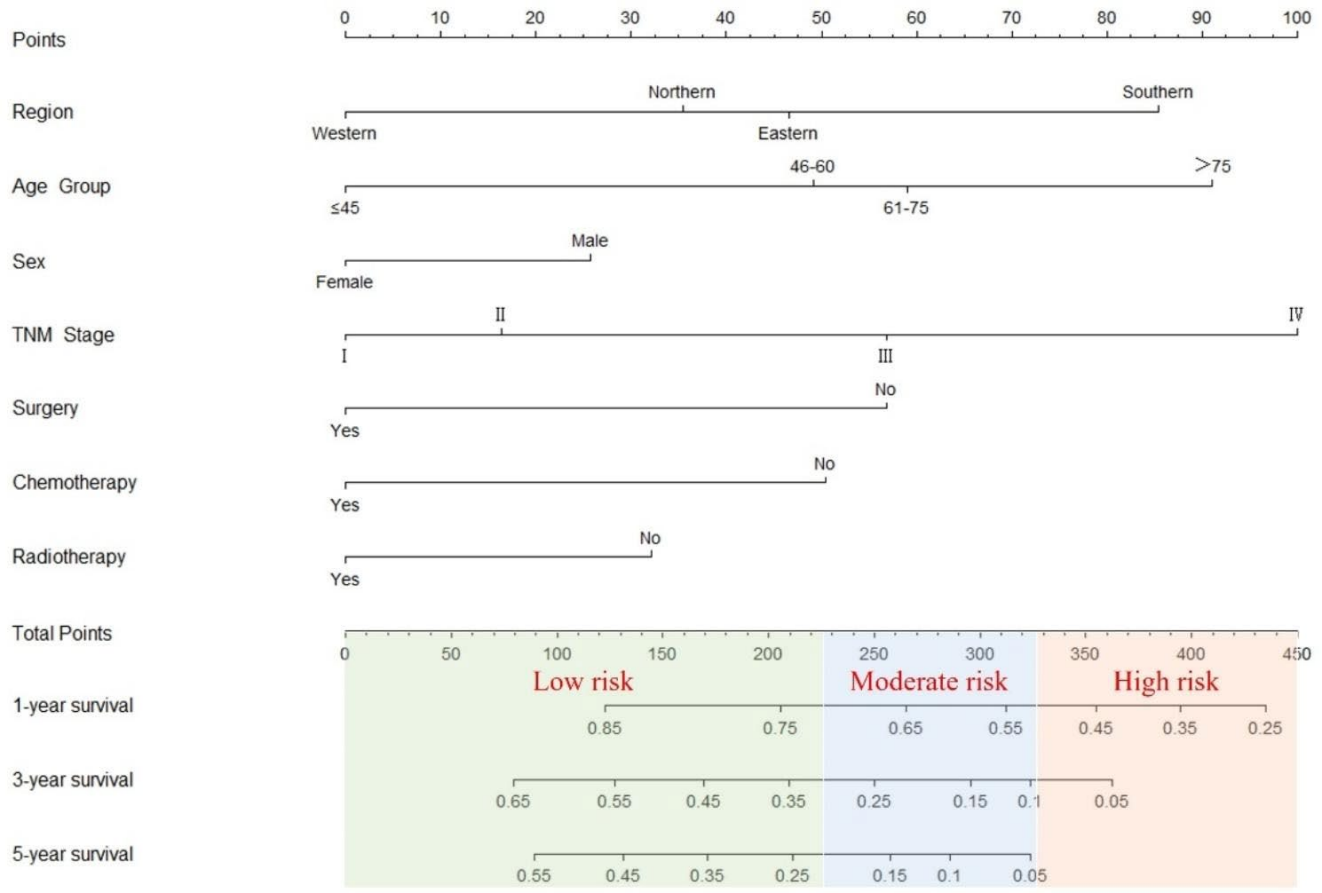
Nomogram predicting 1-year, 3-year, and 5- years OS of patients with small cell lung cancer. *Notes*: Eastern, Southern, Western, and Northern represent respectively Eastern of Shandong, Southern of Shandong, Western of Shandong, and Northern of Shandong

We included region (47 for eastern; 85 for southern; 35 for northern), age (49 for population aged 46–60 years; 59 for population aged 61–75 years; and 91 for population aged > 75 years), sex (26 for male), stage (16 for stage II, 57 for stage III, and 100 for stage IV), surgery (57 for no surgery), chemotherapy (50 for no chemotherapy), and radiotherapy (32 for no radiotherapy) to construct the nomogram prognostic model. To determine a patient’s total score, it was sufficient to sum the above scores for each prognostic factor, and the total score could be used to obtain 1-, 3-, and 5-year survival probability, which can be used as a criterion for evaluating a patient’s prognosis (Fig. 3).

**Fig. 4:**
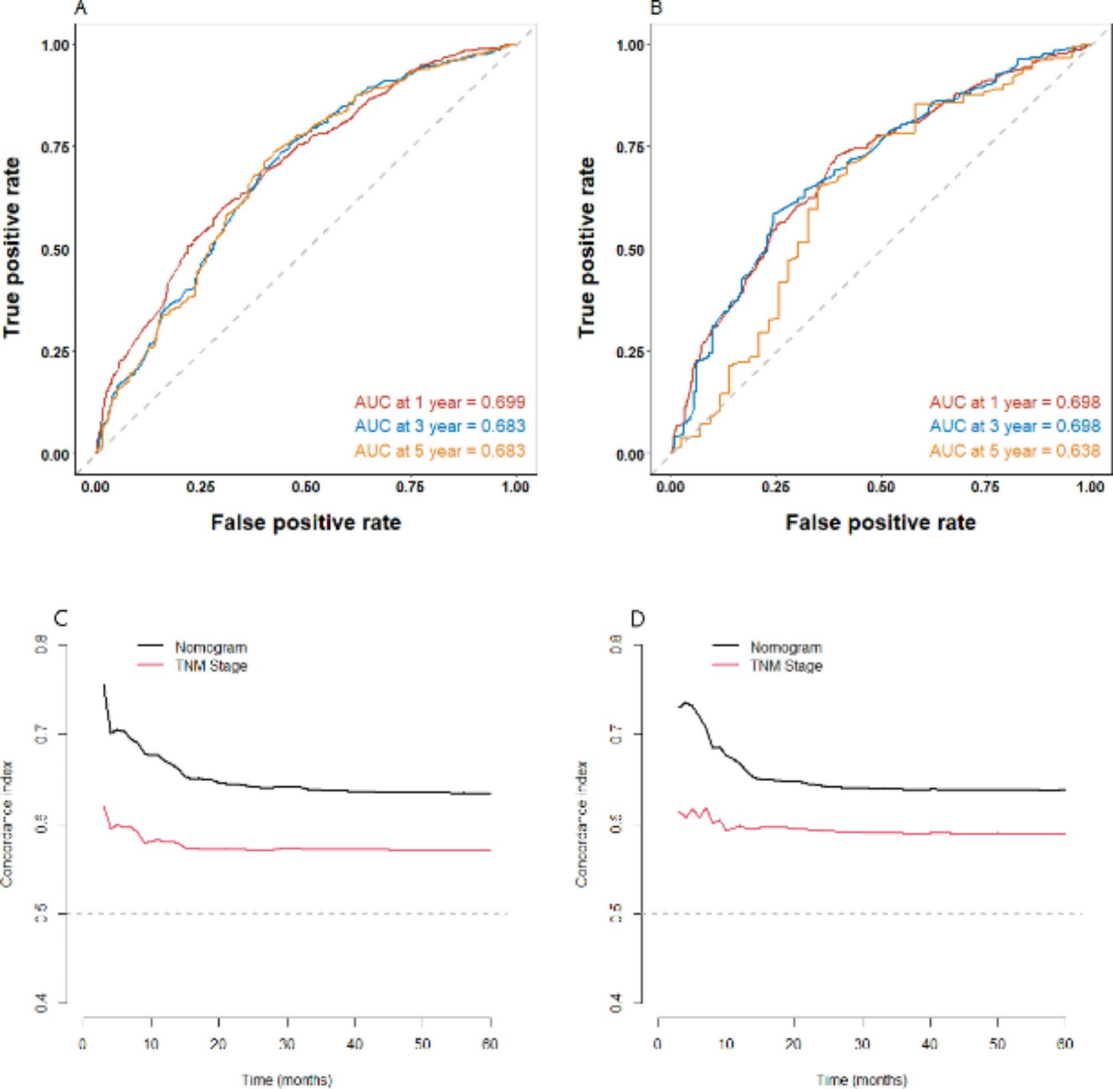
Time-dependent ROC curves of 1-, 3-, and 5-year survival for patients with small cell lung cancer in the training (**A**) and validation (**B**) sets. Time-dependent C-index of 1-, 3-, and 5-year survival for patients with small cell lung cancer in the training (**C**) and validation (**D**) sets. AUC and C-index > 0.6 were considered ideal for the discrimination

The integrating C-index value was 0.634 [95% confidence interval (CI) = 0.610–0.659] in the training set and 0.638 (95% CI = 0.614–0.663) in the validation set (Fig. 4). In the training set, the AUC values were 0.699, 0.683, and 0.683 for 1-, 3-, and 5-year OS, respectively; and in the validation set, the AUC values were 0.698, 0.698, and 0.638 for 1-, 3-, and 5-year OS, respectively. The internal validation of the constructed prognostic model was stable and the first three years of performance on the validation set was essentially indistinguishable from the training set, although the model showed diminishing predictive value as the survival time prolonged. These results were sufficient to show the acceptable discrimination and transportability of the model.

**Fig. 5:**
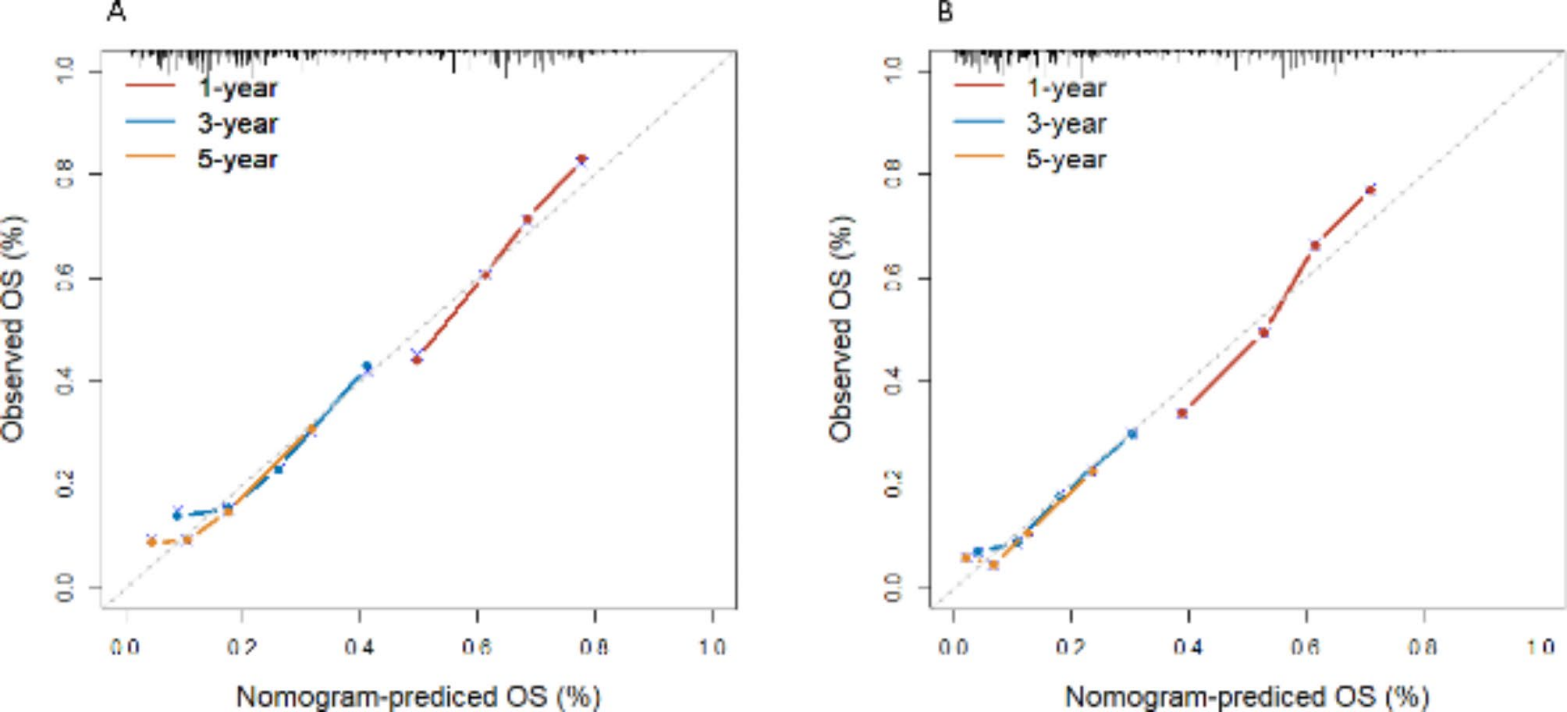
Calibration curves of 1-, 3-, and 5-year overall survival prediction for patients with small cell lung cancer in the training (**A**) and validation (**B**) sets. The closer the calibration curve is to the slash, the more accurate the model predicts survival

To internally validate the nomogram prognostic model, we utilized the Bootstrap method with a self-sampling number of 1,000. Calibration curves were generated for the 1-, 3-, and 5-year survival probability in both the training and validation sets. We found that the calibration curves had a reasonable agreement with the ideal curves, indicating that the difference between the predicted and actual survival probability of patients in the prognostic nomogram was minimal (Fig. 5).

### Clinical usefulness

**Fig. 6:**
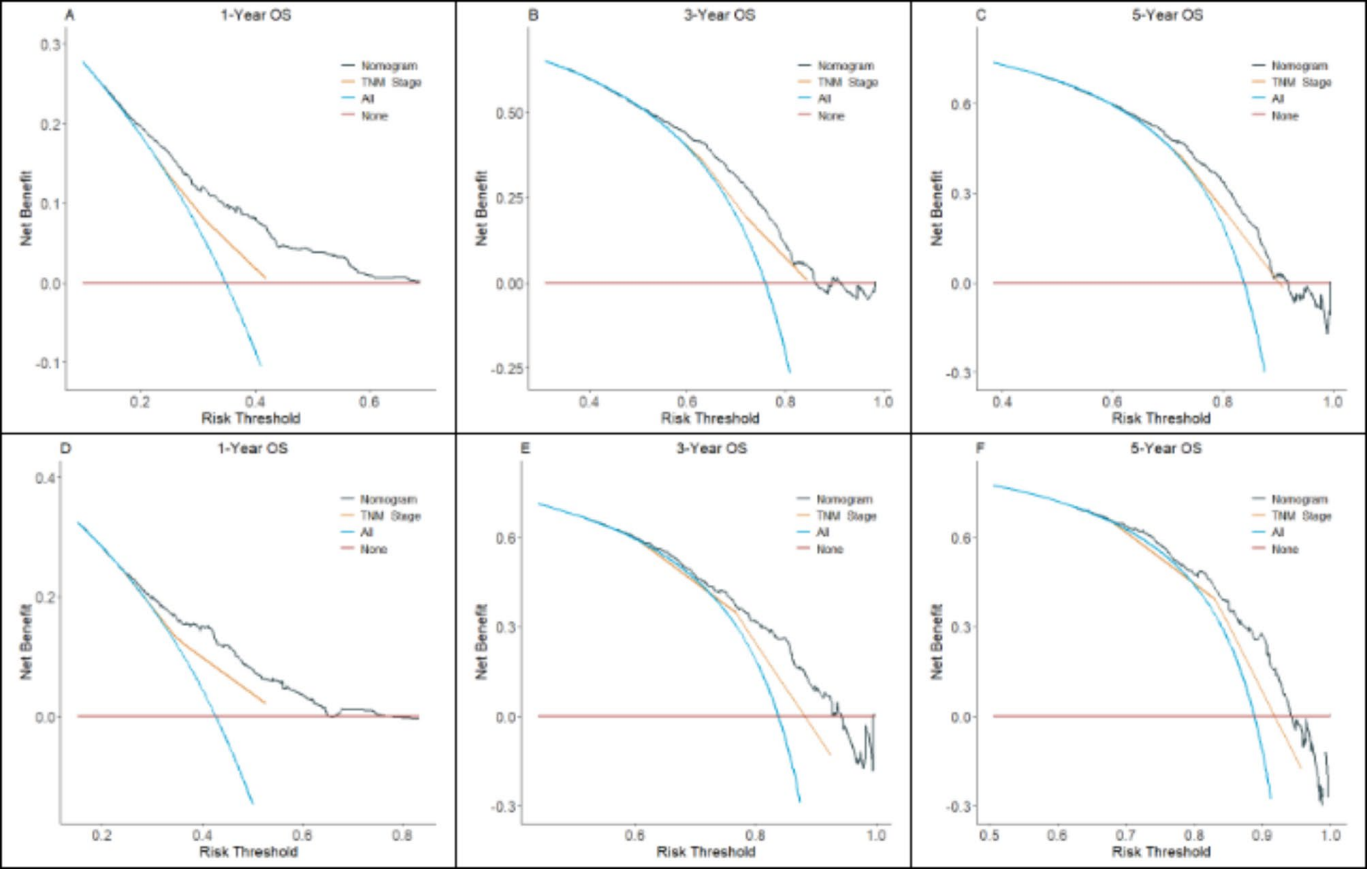
Comparison of DCA between the nomogram and TNM staging system for 1-year (**A**), 3-year (**B**), and 5-year (**C**) in SCLC patients from the training set; comparison of DCA between the nomogram and TNM staging system for 1-year (**D**), 3-year (**E**), and 5-year (**F**) in SCLC patients from the validation set. The x-axis represents the threshold probabilities, and the y-axis represents the net benefit

The DCA plot displays the net benefit of the model compared to two extreme scenarios (treating all patients and treating none) and the TNM staging system. A higher net benefit indicates a more useful model. As shown in Fig. 6, the DCA curves of our constructed prognostic model showed a higher net benefit compared to the two extreme cases and the TNM staging system, proving its clinical value in guiding patients’ treatment decisions.

Changes in NRI and IDI were used to compare the accuracy of the nomogram and the TNM staging system. When nomograms were used in the training cohort, the NRI for 1-, 3-, and 5-year OS was 0.255 (95% CI = 0.189–0.308), 0.210 (95% CI = 0.143–0.274), and 0.198 (95% CI = 0.106–0.274), respectively, and the IDI values for 1-, 3-, and 5-year OS were 0.056 (95% CI = 0.040–0.080), 0.047 (95% CI = 0.032–0.071) and 0.041 (95% CI = 0.024–0.067), respectively (Table 3). These results were validated in the validation cohort (Table 3), and although there was no difference between the two models in predicting 3- and 5-year OS, the nomogram predicted prognosis showed a greater accuracy than the TNM staging system.

**Table 3:** NRI and IDI of the nomogram and the TNM stage in survival prediction for SCLC patients in the training and validation sets

Index	Training set				Validation set		
	Estimate	*95%CI*	*p*		Estimate	*95%CI*	*p*
NRI (vs. the TNM stage)							
1-year OS	0.255	0.189–0.308	< 0.001		0.154	0.034–0.257	0.012
3-year OS	0.210	0.143–0.274	< 0.001		0.157	-0.030-0.271	0.096
5-year OS	0.198	0.106–0.274	< 0.001		-0.040	-0.226-0.106	0.363
IDI (vs. the TNM stage)							
1-year OS	0.056	0.040–0.080	< 0.001		0.040	0.014–0.065	0.004
3-year OS	0.047	0.032–0.071	< 0.001		0.025	-0.010-0.052	0.112
5-year OS	0.041	0.024–0.067	< 0.001		-0.006	-0.064-0.031	0.715

*Note*: NRI, Net Reclassification Improvement; IDI, Integrated Discrimination Improvement; OS, overall survival

### Risk stratification based on the nomogram

**Fig. 7:**
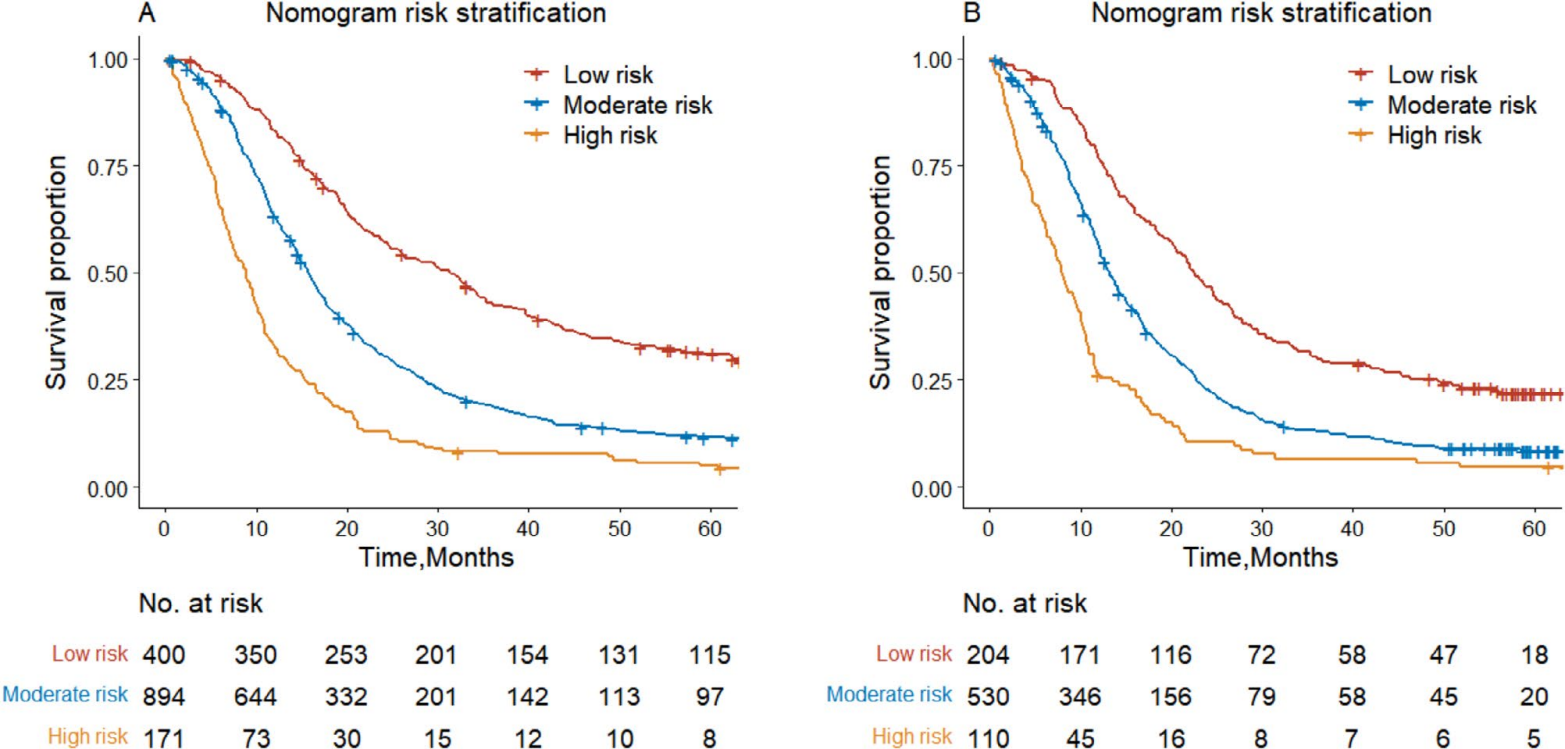
Kaplan-Meier curve analyses categorized by the risk classification system. Risk stratification of SCLC patients’ overall survival in the training (**A**) and validation (**B**) sets

We finally made risk stratifications based on total points calculated using the nomogram and TNM stage. Patients with SCLC were divided into three risk groups [18]: low risk (total points < 226), middle risk (226 ≤ total points < 328), and high risk (total points ≥ 328). The Kaplan-Meier OS curves based on the nomogram showed a great discrimination (Table 4; Fig. 7).

**Table 4:** Cox regression analysis for risk stratification of nomograms in the training and validation sets

	Survival rate (%)			Median survival time (months)	*HR (95% CI)*	*P Value*
	1 – Year	3 – Year	5 – Year			
Training set						
Low risk	83.18	42.82	31.12	31.43	Reference	
Moderate risk	63.24	18.77	11.59	15.70	1.974(1.723–2.263)	< 0.001
High risk	32.75	8.19	5.04	8.83	3.798(3.126–4.614)	< 0.001
Validation set						
Low risk	76.75	29.71	21.91	22.57	Reference	
Moderate risk	56.30	12.94	8.36	13.23	1.785(1.488–2.141)	< 0.001
High risk	26.36	6.59	4.71	7.97	3.154(2.455–4.050)	< 0.001

### Sensitivity analysis

Supplementary material (Supplement 1) includes e Table. 1, which shows univariate Cox regression analyses for the complete and post-screening data, and e Table. 2, which shows sensitivity analyses for age as a continuous and categorical variable. The results of adjusting the variables indicated a high level of consistency between the hazard ratios (HR) before and after adjustment, demonstrating the stability of the nomogram. These findings provided further support for the reliability of the nomogram prognostic model in predicting patient outcomes and guiding treatment decisions.

## Discussion

This retrospective study analyzed 2309 SCLC patients from multiple centers in Shandong Province between 2010 and 2014. The patients were recruited from different general or oncology hospitals in different regions, which ensured their representativeness and universality among Chinese SCLC patients. The nomogram prognostic model in this study enabled intuitive and effective visualization and analysis of the influencing factors. By conducting univariate and multivariate Cox proportional hazard regression analyses to eliminate confounding factors, we identified sex, age, region, TNM stage, surgery, chemotherapy, and radiotherapy as key variables to establish the nomogram.

Nomograms have been shown to be effective predictive tools for assessing patient prognosis [19–21]. In the case of SCLC, most prognostic models have been constructed using data from public databases or hospital medical record systems [11–14]. Liang et al. [8] developed two SCLC nomograms for limited and extensive staging using the SEER database, and the models both outperformed the 8th TNM staging system. However, the variables to be selected for the development of the models did not have surgery status, and the study subjects selected had to be those who had received chemotherapy, and the final model included missing values for radiotherapy, which inevitably reduced the value of the models. Wang et al. [11] used the NCDB database to establish an SCLC nomogram that included seven independent prognostic factors, which were race, age, gender, TNM stage, therapeutic regimen, tumor laterality, and Charlson/Deyo score. It outperformed the two traditional staging systems and had good discrimination with an integrated AUC of 0.789, but it was predictable for 30 months, which was shorter than our model. Pan et al. [12] constructed an SCLC nomogram using data from the First Affiliated Hospital of Guangzhou Medical University, and the C-index of the predictive probability of this model (0.68) was higher than that of TNM staging (0.65). Xiao et al. [13] used patient data from the tertiary cancer hospital affiliated with the Xiang-ya Medical School of Central South University in Changsha, China, and established an SCLC model with a C-index of only 0.60. These two studies did not have sufficient sample sizes or multicenter representative populations, nor were they externally validated.

We pioneered the inclusion of regional factors to construct the nomogram model, which was not available in any previous studies. We divided the fifteen cities in Shandong province into four regions based on their geographical locations, and the atmospheric pollution, economic levels, and medical levels differed among the regions. Supplementary Table 3 shows the number of patients in each region of the training set stratified by TNM stage, with significantly more patients in the northern and western regions than in the eastern and southern regions, and a decreasing number of stage IV patients in the northern, eastern, southern, and western regions sequentially; In addition, the economic and medical levels of the northern and eastern regions were higher than those of the western and southern regions; and the eastern and southern regions are coastal and have the best air quality situation, while the northern region has many heavy industrial cities with poor air quality situations. We also analyzed whether the type of hospital the patient attended and health insurance were associated with prognosis, but these two factors related to the economic and medical level were not included in the final prognostic model, which is inconsistent with previous studies that included health insurance [13]. Considering that the occurrence of lung cancer is closely associated with atmospheric pollution [22–24], there may be an association between the prognosis of SCLC and local air quality. Unfortunately, we were unable to refine the study for the time being, and in the future, we consider deepening the study by including a history of chronic obstructive pulmonary disease, emphysema or chronic bronchitis, and specific air pollution indicators such as concentrations of particulate matter (PM2.5, PM10), nitrogen dioxide (NO_2_) and nitrogen oxides (NOx) [25, 26].

Our model calculated individualized survival probabilities for each patient, which reflected the heterogeneity among them. Although the model has some predictive power, it is not ideal. The AUC area in the validation of the nomogram in this study just reached 0.7, and the calibration curve showed that the actual observations were in some agreement with the nomogram. The DCA curve results demonstrate that clinicians could use it as a reference to predict the progression of each SCLC patient and make clinical decisions. The nomogram prediction model still has a significant reference value in Shandong and even in China.

The lack of specific clinical features makes SCLC screening criteria non-uniform [27], while most patients are diagnosed with extensive-stage SCLC. In this case, surgical treatment is unfeasible. Instead, radiotherapy and chemotherapy are the most commonly used treatment strategies [3, 4, 7]. The patients in this study fit this profile, with over half of the SCLC patients being over 60 years old, and the vast majority of patients being in stage III or IV, receiving chemotherapy and radiotherapy but no surgery. Limited-stage SCLC patients who undergo surgery have a higher survival rate than those with extensive-stage SCLC, the 5-year overall survival rate for stage I SCLC patients who received surgery and chemotherapy was detected to be 49% [28, 29]. The treatment lines of chemotherapy and radiotherapy for SCLC patients from 2010 to 2014 are shown in e Table 4 and e Fig. 1. Among patients treated with chemotherapy, those who received postoperative chemotherapy had the highest survival rate; among patients treated with radiotherapy, sequential chemo-radiotherapy, and simultaneous radio-chemotherapy were the most numerous and had a higher survival rate. In general, the prognosis of patients in 2014 was significantly worse than that of patients in 2010 − 2013 years, and this may be because the number of patients who receive operations, chemotherapy, and radiotherapy was lower in 14 years than in 10 − 13 years, whereas there are more patients in stage IV than in 10 − 13 years (Table 1). Moreover, our model also showed that the risk of death was lower in females than in males, which may be due to the prevalence of smoking, and the amount of stage IV was lower in females than in males in our study (e Table 5).

Additionally, the study only focused on SCLC patients and did not consider other types of lung cancer, which limited the generalizability of the nomogram for other types of lung cancer. Moreover, the study did not account for potential genetic and molecular biomarkers that may affect the prognosis and treatment outcomes of SCLC patients. Future studies should consider integrating such factors to improve the accuracy of the nomogram. Lastly, as a retrospective study, there may be biases and confounding factors that were not accounted for in the analysis. Therefore, caution should be taken when applying the nomogram in clinical practice.

## Conclusion

By retrospectively studying SCLC patients in Shandong province, we developed an SCLC prognostic nomogram, validated it, and proved that the model performed well. The established nomogram model can be used for a more accurate prognosis prediction and reference for therapeutic regimen selection in SCLC patients. However, while the model shows promise for clinical application in SCLC, further studies are needed to determine whether differences in prognosis for SCLC patients in the same province are related to factors such as atmospheric pollution conditions. Additionally, measures such as improving secondary prevention efforts and completeness of follow-up in tumor registry reporting could complete the model. Finally, more opportunities for lung cancer screening should be made available to individuals at high risk. By combining research, prevention, and control efforts, we can work towards reducing the disease burden for SCLC patients.

### Electronic supplementary material

Below is the link to the electronic supplementary material.

**Supplementary Material 1:** **eTable.1:** Univariate Cox regression analyses for the complete and post-screening data in SCLC patients. **eTable.2:** Multivariate Cox regression analyses for the SCLC patients in the training set. **eTable.3:** TNM staging stratification of patients in the region of the training set. **eTable.4:** Characteristics of chemotherapy and radiotherapy treatment lines for all SCLC patients. **e Figure. 1:** Survival curves of chemotherapy and radiotherapy treatment lines for SCLC patients. **eTable.5:** Characteristics of patients stratified by sex in the training set. **eTable.6:** Chemotherapy regimens reference

## Data Availability

All data generated or analyzed during this study are included in this published article and its additional information files.
