# Supplementary material for: Construction and validation of a nomogram to predict the overall survival of small cell lung cancer: a multicenter retrospective study in Shandong province, China
Source: BMC Cancer. 2023 Dec 1;23:1182. doi: 10.1186/s12885-023-11692-7 (PMC10693064; doi:10.1186/s12885-023-11692-7)
Supplement: Supplementary file 1 — Supplementary Material 1: eTable.1: Univariate Cox regression analyses for the complete and post-screening data in SCLC patients. eTable.2: Multivariate Cox regression analyses for the SCLC patients in the training set. eTable.3: TNM staging stratification of patients in the region of the training set. eTable.4: Characteristics of chemotherapy and radiotherapy treatment lines for all SCLC patients. e Figure. 1: Survival curves of chemotherapy and radiotherapy treatment lines for SCLC patients. eTable.5: Characteristics of patients stratified by sex in the training set. eTable.6: Chemotherapy regimens reference [file 12885_2023_11692_MOESM1_ESM.docx]

**Supplement**

eTable.1 Univariate Cox regression analyses for the complete and post-screening data in SCLC patients

eTable.2 Multivariate Cox regression analyses for the SCLC patients in the training set

eTable.3 TNM staging stratification of patients in the region of the training set

eTable.4 Characteristics of chemotherapy and radiotherapy treatment lines for all SCLC patients

e Figure. 1 Survival curves of chemotherapy and radiotherapy treatment lines for SCLC patients

eTable.5 Characteristics of patients stratified by sex in the training set

eTable.6 Chemotherapy regimens reference

eTable.1 Univariate Cox regression analyses for the complete and post-screening data in SCLC patients

| Variables | Complete data | |  | Adjusted data | |
| --- | --- | --- | --- | --- | --- |
|  | HR (95% CI) | *P* Value |  | HR (95% CI) | *P* Value |
| Sex |  |  |  |  |  |
| Male | Reference |  |  | Reference |  |
| Female | 0.806 (0.743-0.875) | < 0.001 |  | 0.827 (0.748-0.914) | < 0.001 |
| Age |  |  |  |  |  |
| ≤45 | Reference |  |  | Reference |  |
| 46-60 | 1.206 (1.028-1.416) | 0.021 |  | 1.365 (1.118-1.665) | 0.002 |
| 61-75 | 1.467 (1.253-1.718) | < 0.001 |  | 1.633 (1.341-1.989) | < 0.001 |
| ＞75 | 2.096 (1.724-2.548) | < 0.001 |  | 2.428 (1.906-3.094) | < 0.001 |
| Smoke |  |  |  |  |  |
| No | Reference |  |  | Reference |  |
| Yes | 1.178 (1.094-1.269) | < 0.001 |  | 1.151 (1.051-1.259) | 0.002 |
| Alcohol use |  |  |  |  |  |
| No | Reference |  |  | Reference |  |
| Yes | 1.039 (0.961-1.124) | 0.339 |  | 1.022 (0.931-1.121) | 0.648 |
| Region |  |  |  |  |  |
| Eastern | Reference |  |  | Reference |  |
| Southern | 1.16 (1.005-1.339) | 0.043 |  | 1.313 (1.11-1.553) | 0.001 |
| Western | 0.864 (0.782-0.954) | 0.004 |  | 0.803 (0.707-0.913) | 0.001 |
| Northern | 0.999 (0.905-1.103) | 0.981 |  | 0.956 (0.845-1.08) | 0.468 |
| Hospital |  |  |  |  |  |
| Specialized | Reference |  |  | Reference |  |
| General | 1.032 (0.924-1.154) | 0.576 |  | 1.03 (0.91-1.165) | 0.642 |
| Health insurance |  |  |  |  |  |
| Urban employees' basic medical insurance | Reference |  |  | Reference |  |
| Urban residents' basic medical insurance | 1.112 (0.979-1.263) | 0.103 |  | 1.058 (0.908-1.231) | 0.472 |
| New rural cooperative medical scheme | 1.09 (0.993-1.196) | 0.069 |  | 1.044 (0.936-1.164) | 0.443 |
| Self-pay | 1.108 (0.895-1.37) | 0.347 |  | 1.141 (0.879-1.48) | 0.322 |
| Other | 0.982 (0.751-1.283) | 0.894 |  | 0.838 (0.621-1.133) | 0.251 |
| TNM Stage |  |  |  |  |  |
| Ⅰ | Reference |  |  | Reference |  |
| Ⅱ | 1.251 (0.991-1.579) | 0.059 |  | 1.282 (0.989-1.662) | 0.061 |
| Ⅲ | 1.511 (1.235-1.848) | < 0.001 |  | 1.536 (1.227-1.922) | < 0.001 |
| Ⅳ | 2.161 (1.769-2.638) | < 0.001 |  | 2.336 (1.87-2.918) | < 0.001 |
| Surgery |  |  |  |  |  |
| No | Reference |  |  | Reference |  |
| Yes | 0.553 (0.467-0.655) | < 0.001 |  | 0.522 (0.424-0.642) | < 0.001 |
| Chemotherapy |  |  |  |  |  |
| No | Reference |  |  | Reference |  |
| Yes | 0.717 (0.659-0.78) | < 0.001 |  | 0.704 (0.634-0.781) | < 0.001 |
| Radiotherapy |  |  |  |  |  |
| No | Reference |  |  | Reference |  |
| Yes | 0.723 (0.659-0.793) | < 0.001 |  | 0.723 (0.651-0.804) | < 0.001 |

Notes: HR, hazard ratio; Eastern, Southern, Western, and Northern represent respectively Eastern of Shandong, Southern of Shandong, Western of Shandong, and Northern of Shandong; Specialized, General represent respectively specialized tumor hospitals and general hospitals.

eTable.2 Multivariate Cox regression analyses for the SCLC patients in the training set

| Variables | | Unadjusted model | |  | Adjusted model | |
| --- | --- | --- | --- | --- | --- | --- |
|  |  | HR (95% CI) | *P* Value |  | HR (95% CI) | *P* Value |
| Sex | |  |  |  |  |  |
| Male | | Reference |  |  | Reference |  |
| Female | | 0.856 (0.731-1.002) | 0.053 |  | 0.856 (0.731-1.003) | 0.054 |
| Age Group | |  |  |  |  |  |
| ≤45 | | Reference |  |  |  |  |
| 46-60 | | 1.388 (1.093-1.763) | 0.007 |  |  |  |
| 61-75 | | 1.496 (1.177-1.901) | 0.001 |  |  |  |
| ＞75 | | 1.883 (1.391-2.55) | < 0.001 |  |  |  |
| Age (continuous variable) (continuous variable |  | |  |  | 1.012 (1.007-1.018) | < 0.001 |
| Smoke | |  |  |  |  |  |
| No | | Reference |  |  | Reference |  |
| Yes | | 0.995 (0.852-1.163) | 0.952 |  | 0.989 (0.846-1.156) | 0.891 |
| Alcohol use | |  |  |  |  |  |
| No | | Reference |  |  | Reference |  |
| Yes | | 1.08 (0.937-1.246) | 0.288 |  | 1.082 (0.939-1.247) | 0.278 |
| Region | |  |  |  |  |  |
| Eastern | | Reference |  |  | Reference |  |
| Southern | | 1.261 (1.004-1.583) | 0.046 |  | 1.237 (0.986-1.551) | 0.066 |
| Western | | 0.699 (0.589-0.83) | < 0.001 |  | 0.699 (0.589-0.829) | < 0.001 |
| Northern | | 0.903 (0.776-1.052) | 0.190 |  | 0.897 (0.771-1.045) | 0.163 |
| Hospital | |  |  |  |  |  |
| Specialized hospital | | Reference |  |  | Reference |  |
| General | | 0.948 (0.801-1.121) | 0.530 |  | 0.941 (0.795-1.113) | 0.475 |
| Health insurance | | Reference |  |  | Reference |  |
| Urban employees' basic medical insurance | | 0.927 (0.756-1.137) | 0.467 |  | 0.922 (0.753-1.13) | 0.437 |
| Urban residents' basic medical insurance | | 1.029 (0.894-1.184) | 0.694 |  | 1.035 (0.9-1.19) | 0.631 |
| New rural cooperative medical scheme | | 0.957 (0.708-1.293) | 0.775 |  | 0.956 (0.708-1.292) | 0.770 |
| Self-pay | | 0.736 (0.436-1.241) | 0.250 |  | 0.737 (0.437-1.242) | 0.252 |
| Other | |  |  |  |  |  |
| TNM Stage | |  |  |  |  |  |
| Ⅰ | | Reference |  |  | Reference |  |
| Ⅱ | | 1.105 (0.789-1.548) | 0.563 |  | 1.118 (0.798-1.567) | 0.517 |
| Ⅲ | | 1.458 (1.083-1.964) | 0.013 |  | 1.459 (1.084-1.965) | 0.013 |
| Ⅳ | | 1.971 (1.463-2.656) | < 0.001 |  | 1.967 (1.461-2.649) | < 0.001 |
| Surgery | |  |  |  |  |  |
| No | | Reference |  |  | Reference |  |
| Yes | | 0.676 (0.519-0.881) | 0.004 |  | 0.683 (0.524-0.889) | 0.005 |
| Chemotherapy | |  |  |  |  |  |
| No | | Reference |  |  | Reference |  |
| Yes | | 0.707 (0.615-0.813) | < 0.001 |  | 0.716 (0.623-0.822) | < 0.001 |
| Radiotherapy | |  |  |  |  |  |
| No | | Reference |  |  | Reference |  |
| Yes | | 0.797 (0.696-0.912) | 0.001 |  | 0.796 (0.695-0.911) | 0.001 |

Notes: Eastern, Southern, Western, and Northern represent respectively Eastern of Shandong, Southern of Shandong, Western of Shandong, and Northern of Shandong; Specialized, General represent respectively specialized tumor hospitals and general hospitals.

eTable.3 TNM staging stratification of patients in the region of the training set

|  | Region | | | | χ^2^ | *P* value |
| --- | --- | --- | --- | --- | --- | --- |
|  | Eastern  (N = 302) | Southern  (N = 122) | Western  (N = 402) | Northern  (N = 639) |  |  |
| Ⅰ | 15(4.97) | 6(4.92) | 20(4.98) | 34(5.32) |  |  |
| Ⅱ | 33(10.93) | 12(9.84) | 38(9.45) | 58(9.08) |  |  |
| Ⅲ | 95(31.46) | 54(44.26) | 193(48.01) | 250(39.12) | 22.928 | 0.006 |
| Ⅳ | 159(52.65) | 50(40.98) | 151(37.56) | 297(46.48) |  |  |

eTable.4 Characteristics of chemotherapy and radiotherapy treatment lines for all SCLC patients

| Characteristics | Censored  (N=326) | Death  (N=1983) | χ^2^ | *P* value |
| --- | --- | --- | --- | --- |
| Chemotherapy treatment line |  |  |  |  |
| no chemotherapy | 57(17.48) | 464(23.40) |  |  |
| pre-surgery | 39(11.96) | 339(17.10) |  |  |
| post-surgery | 26(7.98) | 51(2.57) |  |  |
| pre-radiotherapy | 73(22.39) | 316(15.94) | 45.474 | < 0.001 |
| post-radiotherapy | 0(0.00) | 21(1.06) |  |  |
| simultaneous radiotherapy and chemotherapy | 19(5.83) | 84(4.24) |  |  |
| unknown | 112(34.36) | 708(35.70) |  |  |
| Radiotherapy treatment line |  |  |  |  |
| no radiotherapy | 226(69.33) | 1539(77.61) |  |  |
| pre-surgery | 3(0.92) | 8(0.40) |  |  |
| post-surgery | 2(0.61) | 4(0.20) |  |  |
| pre-chemotherapy | 2(0.61) | 23(1.16) | 23.828 | 0.001 |
| post-chemotherapy | 64(19.63) | 260(13.11) |  |  |
| simultaneous radiotherapy and chemotherapy | 27(8.28) | 103(5.19) |  |  |
| unknown | 2(0.61) | 46(2.32) |  |  |

| 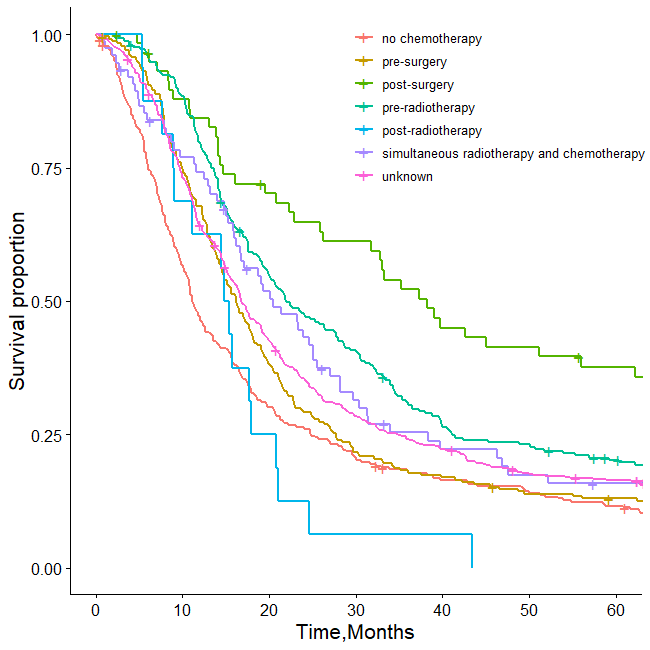 | **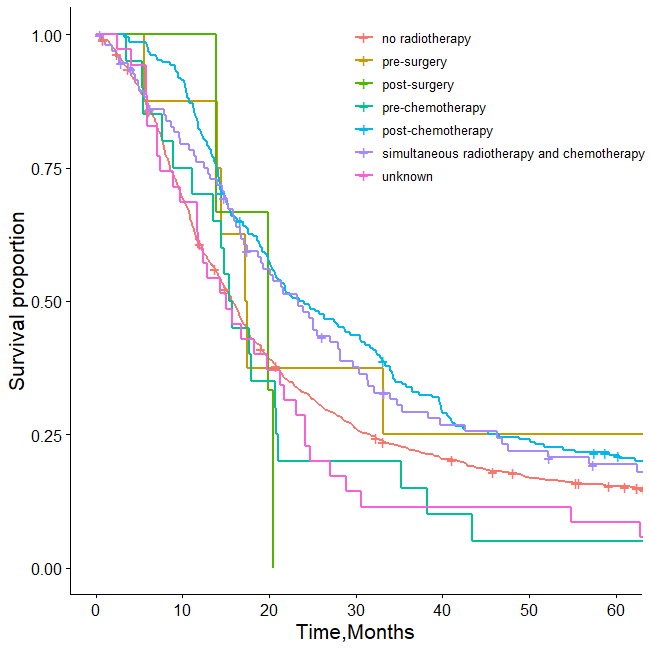** |
| --- | --- |

e Figure. 1 Survival curves of chemotherapy and radiotherapy treatment lines for SCLC patients

eTable.5 Characteristics of patients stratified by sex in the training set

|  | Male  (N = 1071) | Female  (N = 394) | χ^2^ | *P* value |
| --- | --- | --- | --- | --- |
| Smoke |  |  |  |  |
| No | 238(22.22) | 344(87.31) |  |  |
| Yes | 833(77.78) | 50(12.69) | 509.605 | < 0.001 |
| TNM Stage |  |  |  |  |
| Ⅰ | 53(4.95) | 22(5.58) |  |  |
| Ⅱ | 98(9.15) | 43(10.91) |  |  |
| Ⅲ | 431(40.24) | 161(40.86) | 1.770 | 0.621 |
| Ⅳ | 489(45.66) | 168(42.64) |  |  |

eTable.6 Chemotherapy regimens reference

|  | Chemotherapy Regimens |
| --- | --- |
| limited-stage small-cell lung cancer | Etoposide + Cisplatin |
|  | Etoposide + Carboplatin |
|  |  |
| extensive-stage small-cell lung cancer | Etoposide + Carboplatin + Atelizumab |
|  | Etoposide + Platinum + Duvalizumab |
|  | Etoposide + Platinum + Adelbilimab |
|  | Etoposide + Platinum + Sluritumab |
|  | Etoposide + Platinum |
|  | Etoposide + Cisplatin |
|  | Etoposide + Carboplatin |
|  | Irinotecan + Cisplatin |
|  | Irinotecan + Carboplatin |
|  | Etoposide + Loplatin |
